# Supplementary material for: Distance to Natural Environments, Physical Activity, Sleep, and Body Composition in Women: An Exploratory Analysis
Source: Int J Environ Res Public Health. 2023 Feb 18;20(4):3647. doi: 10.3390/ijerph20043647 (PMC9967458; doi:10.3390/ijerph20043647)
Supplement: Supplementary file 1 [file ijerph-20-03647-s001.zip › ijerph-2141420-supplementary.pdf]

Table S1. Summary of variables of the three sets, which are incorporated in the non-linear canonical correlation analyses.

Variables	Variable type	Categories	Category symbols
		Distance to natural spaces (set 1)	
Distance to green space (GS)	Ordinal	<300<br>≥300	GS<300<br>GS_>/=300
Distance to blue space (BS)	Ordinal	<500<br>≥500	BS<500<br>BS>/=500
		Actigraphy variables (set 2)	
Moderate -vigorous physical activity (MVPA)	Single nominal	MVPA < 150 min/week<br>MVPA ≥ 150 min/week	MVPA_NR<br>MVPA_R
Total sleep time (TST)	Single nominal	TST Non-recommended < 7h<br>TST Recommended ≥ 7h	TST_NR<br>TST_R
Sleep onset latency (SOL)	Single nominal	SOL Non-recommended ≥ 30 min<br>SOL Recommended < 30 min	SL_NR<br>SL_R
		Body composition variables (set 3)	
Fat mass (FM)	Single nominal	FM < 32%<br>FM ≥ 32%	FM_L<br>FM_H
Visceral fat area (VFA)	Single nominal	VFA <100 cm^2<br>VFA ≥ 100 cm^2	VFA_L<br>VFA_H

Table S2. Supplementary data with definitions of green and blue space.

Reference	Definition of green space
**(Maas et et al.,2006)**<br>**(Van den Berg et al., 2010)**	Urban green space, forest, nature conservation areas, and agricultural green space.
**Forman [1]**	Playing fields, wetlands, nature reserves, tree corridors, and market-gardening areas.
**[2]**	Urban green, waterside, forest/woodland, wilderness/type habitat, and country-side/farmland.
**(Akpinar, 2017)**	Urban green space, forest, rangeland, agricultural land, and wetland.
**(Dewulf et al. 2016)**	Agriculture, grassland, forest, swamp, heath land and coastal dune as well as park, recreation, and sport terrains.
**[3]**	Five typological categories: Formal (those with an organized layout and structured path network, and generally well maintained), Informal (those with an informal design and less managed feel), Natural (habitats such as heathland or woodland), Young People's (areas designed for use by children or teenagers), and Sports (areas such as playing fields and tennis courts).
**[4]**	Parks, other open spaces, and agricultural land, excluding domestic gardens.
**[5]**	Publicly accessible areas with natural vegetation, such as grass, plants, or trees [and may include] built environment features, such as urban parks, as well as less managed areas, including woodland and nature reserves.
**[6]**	Parks, fields, forests, conservation areas, lakes, rivers, etc.
**[7]**	Urban green, agricultural green, and forests and natural areas.
**[8]**	'Parks' (e.g., city parks, children's farm), 'recreational area' (e.g., zoo, playground, picnic places), 'agricultural green' (e.g., grassland, orchard), 'forest & moorland' (e.g., forest, moorland, dunes), and 'blue space' (e.g., lakes, rivers, water in parks, seas).
**[9]**	Private and public gardens, parks, cemeteries, zoos, road planting and forests.
**[10]**	Parks (including both undeveloped and developed parks), playgrounds, golf-courses, country clubs, and zoos, and cemeteries.
**Reference**	**Definition of blue space**
**[11]**<br>**[12]**	A visible surface water (e.g., lakes, rivers, canals, coastal water).
**[13]**<br>**[14]**	Outdoor environments – either natural or manmade – that prominently feature water and are accessible to humans either proximally (being in, on or near water) or

**Blue Health** (https://bluehealth2020.eu/)	distally/virtually (being able to see, hear or otherwise sense water)." Examples are coasts, lake, ponds and pond systems, wadis, artificial buffer basins or water courses".
**[15]**	All visible, outdoor, natural surface waters with potential for the promotion of human health and wellbeing. This excludes outdoor swimming pools, garden ponds and fountains, however, it can include modified and artificially constructed spaces that still contain natural surface water such as a canals, dammed lakes, or urban streams/rivers.
**[16]**	Polygon water feature, which included lakes, rivers, tributaries, and large streams. We selected this measure for the analysis because water polygons captured prominent landscape features and waterbodies used for recreation – in contrast with linear water features that included small, less noticeable blue space. We established criteria to increase the likelihood that the water polygons included in the blue space measure were meaningful to the public. For example, we did not include farm ponds or industrial reservoirs.

References

1. Forman, R. *Urban regions: ecology and planning beyond the city*; Cambridge University Press Cambridge: 2008; Volume 408.
2. Barton, J.; Pretty, J. What is the best dose of nature and green exercise for improving mental health? A multi-study analysis. *Environmental science & technology* **2010**, *44*, 3947-3955, doi:10.1021/es903183r.
3. Coombes, E.; Jones, A.; Hillsdon, M. The relationship of physical activity and overweight to objectively measured green space accessibility and use. *Soc. Sci. Med.* **2010**, *70*, 816-822, doi:10.1016/j.socscimed.2009.11.020.
4. Mytton, O.; Townsend, N.; Rutter, H.; Foster, C. Green space and physical activity: An observational study using health survey for England data. *Health & place* **2012**, *18*, 1034-1041, doi:10.1016/j.healthplace.2012.06.003.
5. Lachowycz, K.; Jones, A. Towards a better understanding of the relationship between greenspace and health: Development of a theoretical framework. *Landscape and Urban Planning* **2013**, *118*, 62-69, doi:10.1016/j.landurbplan.2012.10.012.
6. Halonen, J.; Kivimaki, M.; Pentti, J.; Stenholm, S.; Kawachi, I.; Subramanian, S.; Vahtera, J. Green and blue areas as predictors of overweight and obesity in an 8-year follow-up study. *Obesity* **2014**, *22*, 1910-1917, doi:10.1002/oby.20772.
7. Picavet, H.; Milder, I.; Kruize, H.; Vries, S.; Hermans, T.; Wendel-Vos, W. Greener living environment healthier people?: Exploring green space, physical activity and health in the Doetinchem cohort study. *Preventive Medicine* **2016**, *89*, 7-14, doi:10.1016/j.ypmed.2016.04.021.
8. Jansen, F.; Ettema, D.; Kamphuis, C.; Pierik, F.; Dijst, M. How do type and size of natural environments relate to physical activity behavior? *Health Place* **2017**, *46*, 73-81, doi:10.1016/j.healthplace.2017.05.005.
9. Muller, G.; Harhoff, R.; Rahe, C.; Berger, K. Inner-city green space and its association with body mass index and prevalent type 2 diabetes: a cross-sectional study in an urban german city. *BMJ Open* **2018**, *8*, e019062, doi:10.1136/bmjopen-2017-019062.
10. Jimenez, M.; Wellenius, G.; James, P.; Subramanian, S.; Buka, S.; Eaton, C.; Gilman, S.; Loucks, E. Associations of types of green space across the life-course with blood pressure and body mass index. *Environ. Res.* **2020**, *185*, 109411, doi:10.1016/j.envres.2020.109411.
11. Volker, S.; Kistemann, T. The impact of blue space on human health and well-being - Salutogenetic health effects of inland surface waters: a review. *Int J Hyg Environ Health* **2011**, *214*, 449-460, doi:10.1016/j.ijheh.2011.05.001.
12. Gascon, M.; Triguero-Mas, M.; Martínez, D.; Dadvand, P.; Forns, J.; Plasència, A.; Nieuwenhuijsen, M.J. Mental health benefits of long-term exposure to residential green and blue spaces: a systematic review. *Int J Environ Res Public Health* **2015**, *12*, 4354-4379, doi:10.3390/ijerph120404354.
13. Foley, R.; Kistemann, T. Blue space geographies: Enabling health in place. *Health & place* **2015**, *35*, 157-165, doi:https://doi.org/10.1016/j.healthplace.2015.07.003.
14. Grellier, J.; White, M.P.; Albin, M.; Bell, S.; Elliott, L.R.; Gascón, M.; Gualdi, S.; Mancini, L.; Nieuwenhuijsen, M.J.; Sarigiannis, D.A.; et al. BlueHealth: a study programme protocol for mapping and quantifying the potential benefits to public health and well-being from Europe's blue spaces. *BMJ Open* **2017**, *7*, e016188, doi:10.1136/bmjopen-2017-016188.
15. Britton, E.; Kindermann, G.; Domegan, C.; Carlin, C. Blue care: a systematic review of blue space interventions for health and wellbeing. *Health promotion international* **2020**, *35*, 50-69, doi:10.1093/heapro/day103.
16. Poulsen, M.N.; Schwartz, B.S.; Dewalle, J.; Nordberg, C.; Pollak, J.S.; Silva, J.; Mercado, C.I.; Rolka, D.B.; Siegel, K.R.; Hirsch, A.G. Proximity to freshwater blue space and type 2 diabetes onset: the importance of historical and economic context. *Landsc Urban Plan* **2021**, *209*, doi:10.1016/j.landurbplan.2021.104060.
